# Novel risk score model for non-proliferative diabetic retinopathy based on untargeted metabolomics of venous blood

**DOI:** 10.3389/fendo.2023.1180415

**Published:** 2023-08-21

**Authors:** Xinyu Wang, Shu Yang, Guangyan Yang, Jialong Lin, Pengfei Zhao, Jingyun Ding, Hongyan Sun, Ting Meng, Ming Ming Yang, Lin Kang, Zhen Liang

**Affiliations:** ^1^ Department of Geriatrics, Shenzhen People’s Hospital (The Second Clinical Medical College, Jinan University, The First Affiliated Hospital, Southern University of Science and Technology, Shenzhen, China; ^2^ Department of Nephrology, The First People's Hospital of Yunnan Province, The Affiliated Hospital of Kunming University of Science and Technology, Kunming, China; ^3^ Guangdong Provincial Clinical Research Center for Geriatrics, Shenzhen Clinical Research Center for Geriatrics, Shenzhen People’s Hospital (The Second Clinical Medical College, Jinan University, Shenzhen, China; ^4^ The First Affiliated Hospital, Southern University of Science and Technology), Shenzhen, China; ^5^ Department of Cardiovascular Medicine, The Fourth Affiliated Hospital of Guangzhou Medical University, Zengcheng District People’s Hospital of Guangzhou, Guangzhou, China; ^6^ Department of Geriatric, Shenzhen Second People’s Hospital, First Affiliated Hospital of Shenzhen University, Shenzhen, China; ^7^ Department of Ophthalmology, Shenzhen People’s Hospital, The Second Clinical Medical College, Jinan University, The First Affiliated Hospital, Southern University of Science and Technology, Shenzhen, China; ^8^ The Biobank of National Innovation Center for Advanced Medical Devices, Shenzhen People’s Hospital, Shenzhen, China

**Keywords:** nonproliferative diabetic retinopathy (NPDR), diabetic retinopathy (DR), venous blood, untargeted metabolomics, risk score

## Abstract

**Background and Purpose:**

Nonproliferative diabetic retinopathy (NPDR) occurs in the early stages of Diabetic retinopathy (DR), and the study of its metabolic markers will help to prevent DR. Hence, we aimed to establish a risk score based on multiple metabolites through untargeted metabolomic analysis of venous blood from NPDR patients and diabetic non-DR patients.

**Experimental Approach:**

Untargeted metabolomics of venous blood samples from patients with NPDR, diabetes melitus without DR were performed using high-performance liquid chromatography-mass spectrometry.

**Results:**

Detailed metabolomic evaluation showed distinct clusters of metabolites in plasma samples from patients with NPDR and diabetic non-DR patients. NPDR patients had significantly higher levels of phenylacetylglycine, L-aspartic acid, tiglylglycine, and 3-sulfinato-L-alaninate, and lower level of indolelactic acid, threonic acid, L-arginine (Arg), and 4-dodecylbenzenesulfonic acid compared to control. The expression profiles of these eight NPDR risk-related characteristic metabolites were analyzed using Cox regression to establish a risk score model. Subsequently, univariate and multivariate Cox regression analyses were used to determine that this risk score model was a predictor of independent prognosis for NPDR.

**Conclusions:**

Untargeted metabolome analysis of blood metabolites revealed unreported metabolic alterations in NPDR patients compared with those in diabetic non-DR patients or MH. In the venous blood, we identified depleted metabolites thA and Arg, indicating that they might play a role in NPDR development.

## 1 Introduction

With socioeconomic development and lifestyle changes, the incidence of diabetes is increasing yearly. There are 462 million people with diabetes worldwide, and more than 100 million people in China have diabetes (1). Diabetic retinopathy (DR) is one of the most common microvascular complications of diabetes and the leading cause of vision loss and blindness worldwide (2). Identifying the risk factors or markers associated with DR can help identify people at risk for DR and is essential for the early diagnosis and treatment of the disease. Nonproliferative diabetic retinopathy (NPDR) is often overlooked as an early stage of DR owing to a lack of significant symptoms (3). A meta-analysis summarizing studies on the prevalence and risk factors of DR in China from 1990 -2017 found that among people with diabetes, the prevalence of DR was 18.45% (4). DR not only affects the quality of life of patients, but also a risk factor for increased all-cause mortality, vascular mortality and non-cancer mortality (5).

Identifying markers of NPDR facilitates disease screening and the prevention of progression to proliferative diabetic retinopathy (PDR). The main risk factors for DR include hyperglycemia or significant blood glucose fluctuations, hypertension, hyperlipidemia, prolonged diabetes, diabetic kidney disease, pregnancy, and obesity (6, 7). However, these features cannot reveal DR pathogenesis. To date, there are no recognized biomarkers with high specificity and diagnostic efficiency for NPDR.

Growing evidence has shown that metabolomics can reflect the pathophysiological processes of diseases and facilitate the exploration of their mechanisms (8). The discovery of tumor metabolites, including l-2-hydroxyglutaric acid, cystathionine, hypotaurine, sarcosine, and several secondary bile acids, which may induce hypermethylation, modify key signaling proteins, prevent apoptosis, and induce metabolic reprogramming, has renewed interest in cancer metabolism (8). Metabolomics can be divided into non-targeted and targeted metabolomics based on the coverage of metabolites (9, 10). Untargeted metabolomics aims to detect all measurable metabolites and, therefore, has a broader coverage of substances (11). DR development is closely related to metabolic disorders, and correcting metabolic disorders improves DR status (11). These studies support the essential role of metabolites in the development and progression of DR.

DR is a complex metabolism-related disease for which one or a few metabolites are insufficient to assess the risk. In contrast to PDR, NPDR is in the early stages of DR, and the study of its metabolic markers may be more useful for preventing DR. The true primary prevention is to identify people at risk in the absence of NPDR and to prevent them from developing diabetic microangiopathy through interventions. The aim of this study was to develop a risk scoring system in conjunction with metabolomics to characterise individuals according to their risk of developing NPDR in the future.

## 2 Methods

### 2.1 Plasma collection from NPDR and diabetic non-DR patients

We enrolled patients with a history of type 2 diabetes and divided them into two groups based on the presence or absence of comorbid NPDR, while excluding patients with a history of malignancy, active hepatitis, and HIV. Diabetic NPDR patients (n = 70) and diabetic non-DR patients (n = 71) were included as a control group. Clinical data, such as sex, age, and duration of diabetes, were routinely collected from all participants. Blood samples were collected in K2-ethylenediaminetetraacetic acid tubes and centrifuged at 3000 g for 10 min (4°C). The plasma was separated from whole blood, and the final plasma was stored at -80°C. Ophthalmic screening was performed at Shenzhen People’s Hospital, where NPDR was diagnosed using dilated fundus photography and classified into three levels of mild to moderate severity according to the International Clinical Classification of DR. The experiments were approved by the Institutional Review Board and the Ethics Committee of the Shenzhen People’s Hospital (approval no. LL-KT-2018338). Informed written consent was obtained from all participants prior to inclusion in the study.

### 2.2 Sample preparation

Frozen plasma was thawed at 4°C before the metabolomic analysis. We then added a mixture of acetonitrile and methanol to the plasma (100 μL) in 1.5 mL tubes to precipitate the proteins. The mixture was vortexed for 60 s and left to stand for 10 min, followed by centrifugation of the samples at 12,000 rpm for 10 min at 4°C. The supernatant was evaporated to dryness using a concentrator. Finally, the residue was resuspended in 100 μL of mobile phase before liquid chromatography-mass spectrometry (LC-MS) analysis. Quality control (QC) samples were prepared by mixing the same amount of plasma for each sample and using the same procedure to extract the metabolites as the test samples. QC samples constituted approximately 20% of the total sample.

### 2.3 LC-MS analysis

The target compounds were separated on a Waters ACQUITY UPLC BEH Amide (2.1 mm × 100 mm, 1.7 μm) liquid chromatography column using a Vanquish (Thermo Fisher Scientific) ultra-performance liquid chromatograph. The A phase of the liquid chromatography was aqueous, containing 25 mmol/L ammonium acetate and 25 mmol/L ammonia, and the B phase was acetonitrile. The separation was performed on a gradient elution: 0‒0.5 min, 95% B; 0.5‒7 min, 95%‒65% B; 7‒8 min, 65%‒40% B; 8‒9 min, 40% B; 9‒9.1 min, 40%‒95% B; 9.1‒12 min, 95% B. The mobile phase flow rate was 0.5 mL/min, the column temperature was 25°C, and the sample tray temperature was 4 °C. The Thermo Q Exactive HFX mass spectrometer is capable of primary and secondary mass spectrometry data acquisition under the control of the control software (Xcalibur, Thermo). The detailed parameters are as follows: Sheath gas flow rate: 50 Arb, Aux gas flow rate:10 Arb, Capillary temperature: 320°C, Full MS resolution: 60000, MS/MS resolution: 7500, Collision energy: 10/30/60 in NCE mode, Spray Voltage: 3.5 kV (positive), or -3.2 kV (negative).

### 2.4 Identification of metabolites

Raw data were converted to mzXML format using the ProteoWizard software and processed for peak identification, peak extraction, peak alignment, and integration using the R package (kernel XCMS). Subsequently, it was matched to the secondary mass spectrometry database of BiotreeDB (V2.1) for substance annotation, with the algorithm scoring cutoff value set to 0.3.

### 2.5 Data analysis

#### 2.5.1 Metabolomics analysis

The ionization source of the high-resolution mass spectrometry platform was electrospray ionization, with positive and negative ionization modes. In this study, the data in positive ion mode were used for analysis.

#### 2.5.2 Raw data processing

Individual peaks were filtered to eliminate noise. The filtering of deviations was based on relative standard deviation. Regarding the filtering of individual peaks, only peak area data with no more than 50% null values in a single group or no more than 50% null values in all groups were retained. Regarding the simulation of missing values in the raw data, numerical simulation methods were used for a minimum value of one-half. Data were normalized using internal standards.

#### 2.5.3 Principal component analysis

PCA is a statistical method for transforming a set of observed potentially correlated variables into linearly uncorrelated variables (i.e., principal components) through an orthogonal transformation (12). PCA can reveal the internal structure of the data and, thus, better explain the data variables. Metabolomic data can be considered a multivariate data set that can be visualized in a high-dimensional data space coordinate system. Subsequently, PCA can provide a relatively low-dimensional image (two or three-dimensional), presented as a ‘projection’ of the original object at the point containing the most information, effectively using a small number of principal components to reduce the dimensionality of the data using a small number of principal components. Using the SIMCA software (V15.0.2, Sartorius Stedim Data Analytics AB, Umea, Sweden), the data were log-transformed, centrally formatted, and analyzed by automated modeling.

#### 2.5.4 Orthogonal projections to latent structures-discriminant analysis

Metabolomics data based on high-resolution mass spectrometry platforms are high-dimensional (detects many metabolite species) and small-sample (detects small sample size) in nature. In addition, they contain categorical variables and a large number of non-differential variables that may be correlated with each other. Therefore, when we use the PCA model for analysis, the different variables are spread over more principal components owing to the influence of the correlated variables, preventing better visualization and subsequent analysis. Therefore, we used the OPLS-DA statistical method to analyze the results. The OPLS-DA analysis allows us to filter out orthogonal variables in metabolites that are not correlated with categorical variables and to analyze non-orthogonal and orthogonal variables separately, thus obtaining more reliable information on the degree of correlation between-group differences in metabolites and experimental groups. The data were log-transformed plus UV-formatted using the SIMCA software (V15.0.2, Sartorius Stedim Data Analytics AB, Umea, Sweden). First, OPLS-DA modeling analysis was performed on the first principal component, and the quality of the model was tested with 7-fold cross-validation. Subsequently, the cross-validated R Y (interpretability of the model for the categorical variable Y) and Q (predictability of the model), and the validity of the model was further tested by a permutation test in which the order of the categorical variable Y was randomly changed several times to obtain different random Q values.

#### 2.5.5 OPLS-DA permutation test

The permutation test builds the corresponding OPLS-DA model by randomly changing the order of the categorical variable Y several times (n = 200) to obtain the R and Q values of the random model, which is essential to avoid overfitting and assess the statistical significance of the model.

#### 2.5.6 Analysis of variance

The R software (version 4.1.0, http://r-project.org/) was applied for data analysis and plotting. The “limma” package was used to screen for differential metabolites, followed by the “ggplot2” package to plot the heat and volcano maps of differential metabolites (13).

#### 2.5.7 Signaling pathway analysis

In this study, we evaluated different chemical metabolites for pathway analysis and visualization using the Metabolic Analysis website (http://www.metaboanalyst.ca/) (14).

#### 2.5.8 Metabolite screening

The Least Absolute Shrinkage and Selection Operator (LASSO) method is suitable for reducing high-dimensional data. It was used to select the best predictive features for NPDR patients (15). LASSO is performed *via* the “glmnet” package (16). The subject operating characteristic (ROC) curve is useful for evaluating diagnostic performance. The area under the curve (AUC) for single or multiple factors was calculated using the “pROC” software package (17).

#### 2.5.9 Correlation between metabolites

Spearman’s correlation analysis between metabolites was performed using the “ggstatsplot” package, and the results were subsequently visualized using the “ggplot2” package (18).

#### 2.5.10 Prognostic analysis of metabolites

Kaplan‒Meier (KM) curves were constructed to illustrate the probability of NPDR occurring at a given period, and log-rank tests were used to determine differences between groups. The prognostic value of the diagnostic markers was assessed using univariate and multivariate Cox proportional hazards models. KM curves were calculated and plotted using the ‘survival’ and ‘survminer’ software packages (19, 20).

#### 2.5.11 Risk score

In this study, patients’ risk score was calculated based on the normalized expression levels of each metabolite and their corresponding regression coefficients. The risk score was calculated as follows: risk score = coefficient 1 × metabolite 1 expression + coefficient 2 × metabolite 2 expression + coefficient 3 × metabolite 3 expression + coefficient N × metabolite N expression. Patients were divided into high- and low-risk groups according to the median risk score.

## 3 Result

### 3.1 Metabolomic profile of NPDR and diabetes melitus without DR

**Figure 1:**
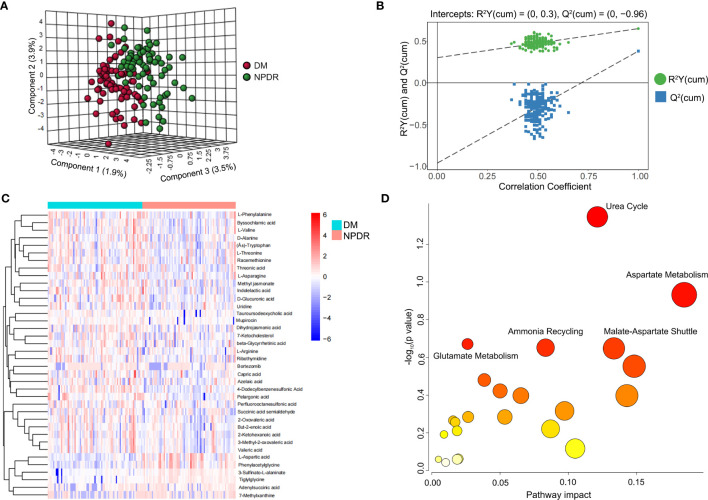
Metabolite characteristics of NPDR and DM patients without DR. **(A)** horizontal coordinates indicate the predicted principal component scores of the first principal component, vertical coordinates indicate the orthogonal principal component scores, and the color of the scatter indicates the different groupings. **(B)** horizontal coordinates indicate the permutation retention of the permutation test (the proportion of the Y variables in the same order as the original model, the points where the permutation retention equals 1 are the R^2^Y and Q^2^ values of the original model), the vertical coordinates indicate the values of R^2^Y or Q^2^, the green dots indicate the R^2^Y values from the permutation test, the blue squares indicate the Q^2^ values from the permutation test, and the two dashed lines indicate the regression lines for R^2^Y and Q^2^ respectively. **(C)** Heat map showing the 38 metabolites differentially expressed in the NPDR (*P*<0.05 by Benjamini & Hochberg). Only urea cycles had a *P*-value <0.05 by Benjamini & Hochberg. **(D)** Enrichment of differential metabolites in the signaling pathway in NPDR, where only the urea cycle had a *P*-value less than 0.05 by Hypergeometric Text.

The scatter plot of OPLS-DA scores showed that the NPDR and diabetic non-DR patient (DM without DR) groups were more significantly differentiated, with samples largely within the 95% confidence interval (CI) **(** **Figure 1A**; **Table 1** **)**. The R^2^Y of the original model was greater than 0.5, indicating that the model established is more consistent with the real situation of the sample data; the Q2 values of the random model of the replacement test were all smaller than the Q2 values of the original model; and the intercept between the regression line of Q2 and the vertical axis was less than zero. At the same time, as the replacement retention gradually decreases, the proportion of the replaced Y variables increases, and the Q2 of the random model gradually decreases, indicating that the original model is robust with no overfitting**(** **Figure 1B** **)**. The heat map demonstrated the 38 plasma metabolites that were differentially expressed in NPDR patients (*P*<0.05) **(** **Figure 1C** and EXCEL EV1). We demonstrated the enrichment of these metabolites in signaling pathways, with the top five ranked according to *P* value being: urea cycle, aspartate metabolism, malate-aspartate shuttle, ammonia recycling, and glutamate metabolism **(** **Figure 1D** **)**.

**Table 1:** Clinical characteristics of NPDR and DM without DR in blood samples.

Parameters	DM without DR patients (n=71)	NPDR patients (n=70)	*P* value
**Sex**	Male : Female=47:24	Male : Female=46:24	NS
**Age**	59.9 ± 9.6	60.7 ± 10.4	NS
**Duration of diabetes (y)**	11.7 ± 6.8	12.4 ± 7.5	NS

Data are calculated by unpaired Student’s t test and presented as mean ± SD. The number of patients in each group was as indicated. NS, not significant.

**Figure 2:**
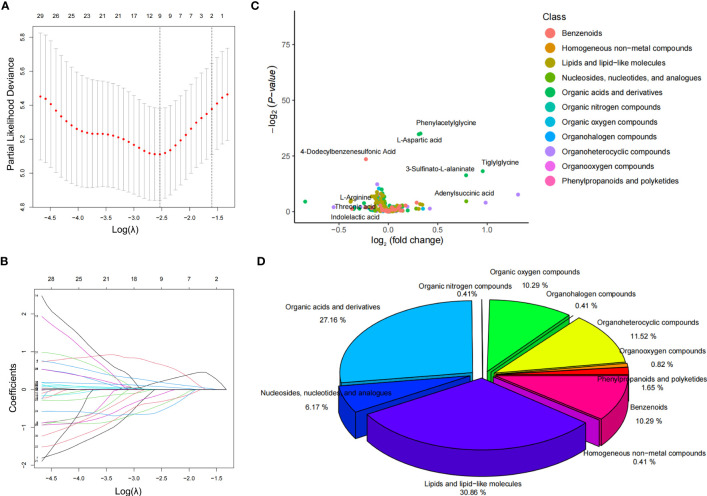
Screening of characteristic metabolites and distribution of metabolites. **(A, B)** LASSO coefficient curves for 38 features. The coefficient curve plots were produced for the log(λ) sequence. A vertical line was drawn at the value selected using the five-fold cross-validation method, where the best λ resulted in coefficients for nine features that were not 0. The best parameter (λ) selection for the LASSO model was cross-validated five-fold by the minimum criterion. Partial likelihood deviation (binomial deviation) curves were plotted against log(λ). Dashed vertical lines were drawn at the optimal values by using the minimum criterion and the 1SE of the minimum criterion (1-SE criterion). **(C)** volcano plot of the distribution of the nine characteristic metabolites in the NPDR blood samples. **(D)** percentage of all metabolite classes that could be identified in the NPDR.

The LASSO method was used to reduce the dimensionality of the data and select metabolite characteristics of NPRD patients **(** **Figures 2A, B** **)**. Nine screened metabolites were distributed in the volcano plot, with four being downregulated and five upregulated **(** **Figure 2C** **)**. Among all metabolite categories that could be identified, the top five were lipids and lipid-like molecules, organic acids and derivatives, organoheterocyclic compounds, organic oxygen compounds, and benzenoids **(** **Figure 2D** **)**.

### 3.2 KM analysis of the characteristic metabolites

**Figure 3:**
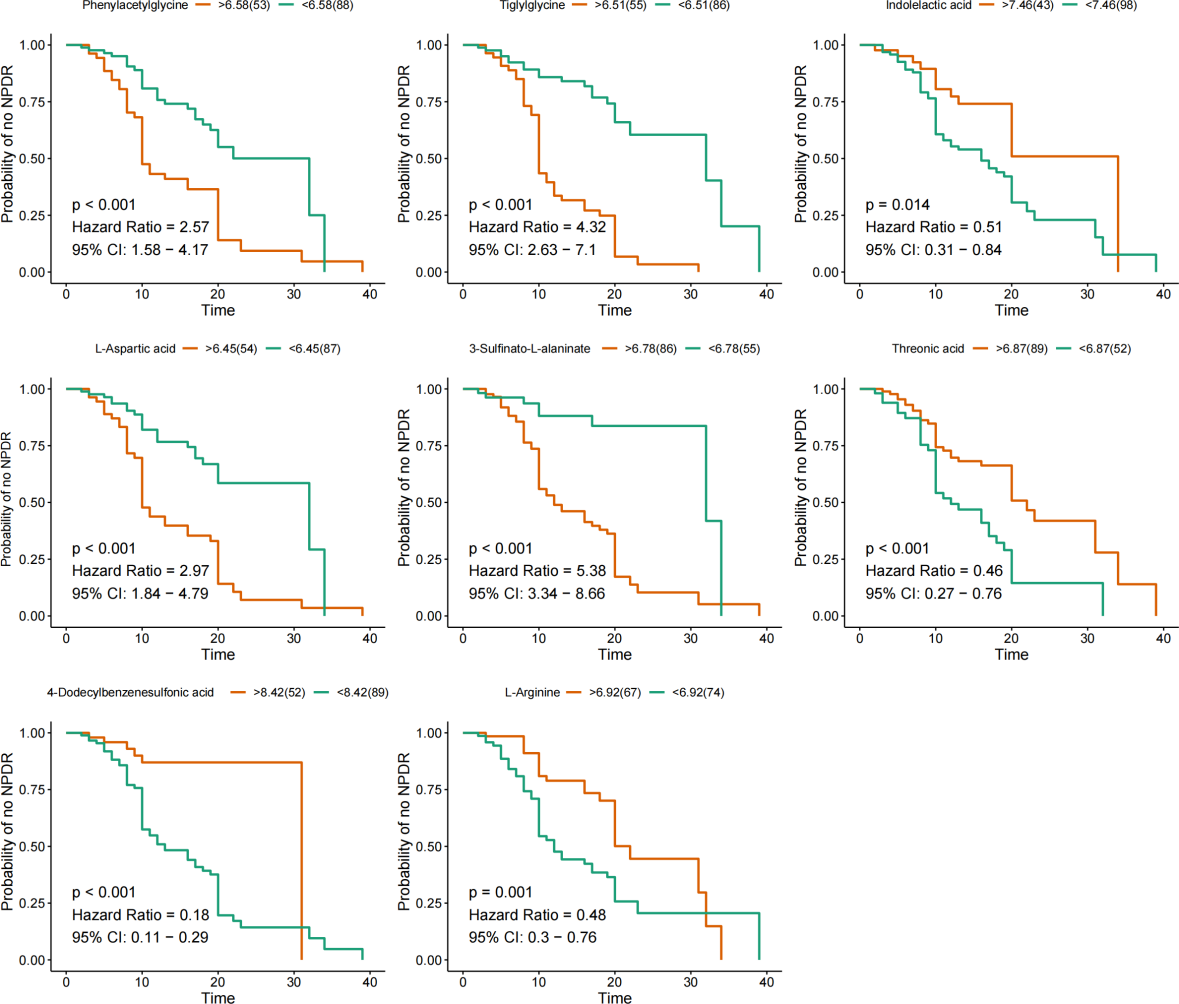
KM curves for characteristic metabolites. KM curves are based on nine metabolites. Hazard ratio (HR) and 95% confidence intervals (CI) for metabolites determined by univariate Cox regression. Time units are years.

The KM method was used to analyze the probability of NPDR occurrence in the corresponding period, and the variables included were nine characteristic metabolites screened by LASSO regression. Metabolites significantly associated with a negative prognosis using NPDR as the endpoint event included phenylacetylglycine, tiglylglycine, L-aspartic acid, and 3-sulfinato-L-alaninate, while indolelactic acid, threonic acid, 4-dodecylbenzenesulfonic acid, and L-arginine were elevated, suggesting a good prognosis **(** **Figure 3** **)**. However, adenylsuccinic acid was not associated with NPDR risk.

### 3.3 Temporal diagnostic validity of characteristic metabolites associated with NPDR risk

**Figure 4:**
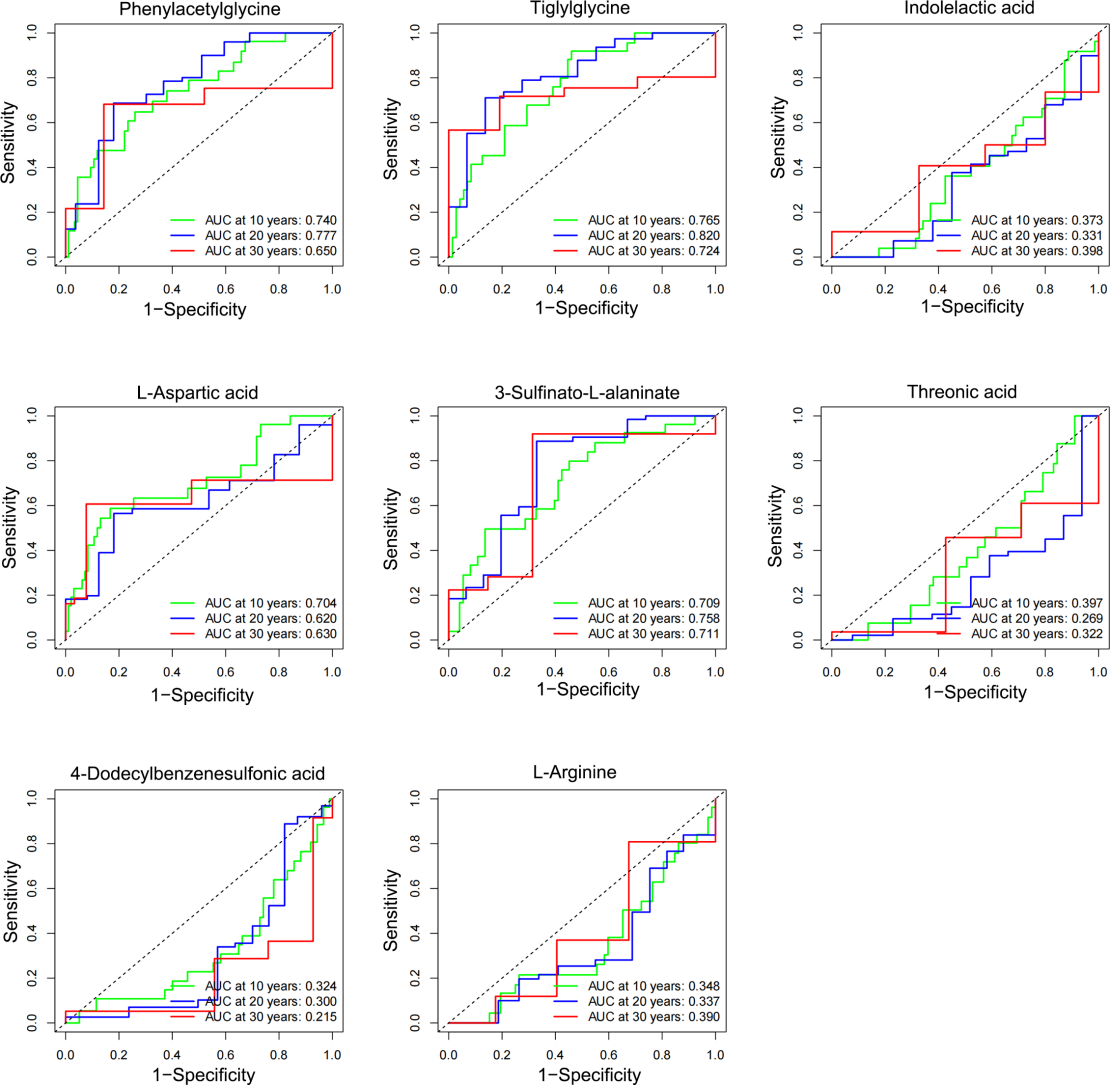
Temporal diagnostic potency of the characteristic metabolites associated with NPDR. The horizontal coordinate represents: 1-specificity, and the vertical coordinate represents: sensitivity. When the AUC was > 0.5, higher values were associated with greater diagnostic potency. When AUC was < 0.5, the lower the value, the stronger the diagnostic potency, and when AUC = 0.5, there was no diagnostic potency.

The eight characteristic metabolites associated with NPDR prognosis were plotted as time-to-subject ROC curves. Consistent with the KM curves, phenylacetylglycine, tiglylglycine, L-aspartic acid, and 3-sulfinato-L-alaninate positively predicted NPDR, whereas indolelactic acid, threonic acid, 4- dodecylbenzenesulfonic acid and L-arginine inversely predicted NPDR. However, of these metabolites, only tiglylglycine showed good diagnostic validity in predicting the risk of NPDR over 20 years (AUC = 0.82) **(** **Figure 4** **)**.

### 3.4 Differential expression and correlation analysis of characteristic metabolites associated with NPDR risk

**Figure 5:**
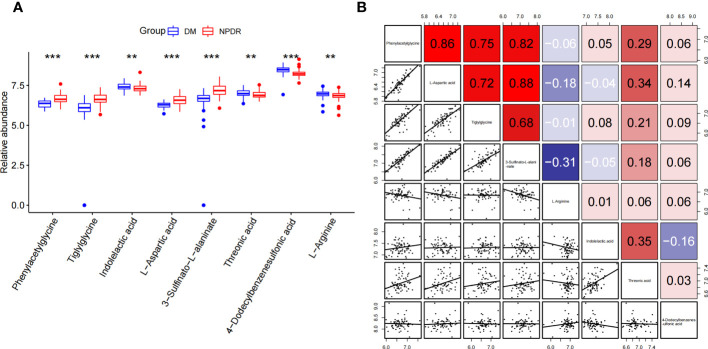
Differential expression and correlation analysis of characteristic metabolites associated with NPDR risk. **(A)** Differential expression of metabolites (NPDR vs. DM without DR). **(B)** Spearman correlation analysis between metabolites. (Red represents positive correlation, blue represents negative correlation). **p<0.01, ***p<0.001 by Wilcox Text.

Phenylacetylglycine, tiglylglycine, L-aspartic acid, and 3-sulfinato-L-alaninate were significantly highly expressed in the plasma of NPDR patients, whereas indolelactic acid, threonic acid, 4-dodecylbenzenesulfonic acid, and L-arginine were significantly less expressed in the plasma of NPDR patients **(** **Figure 5A** **)**. Interestingly, there was a strong positive correlation among the four elevated expression signature metabolites **(** **Figure 5B** **)**.

### 3.5 Risk score modeling and prognostic analysis

**Figure 6:**
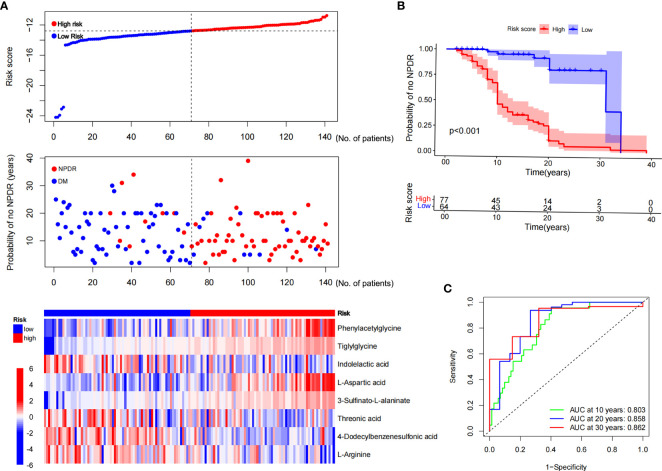
Risk score modeling and prognostic analysis. **(A)** Patients were divided into high- and low-risk groups, using the median risk score as the cutoff value (the upper and middle panel). Heat map consisting of eight characteristic metabolites associated with risk of NPDR (the bottom panel). **(B)** KM curves for patients in the high- and low-risk groups for NPDR. **(C)** AUC of time-dependent ROC curves validating the prognostic performance of the risk score for NPDR.

The expression profiles of the eight NPDR risk-related characteristic metabolites were analyzed using Cox regression to establish a risk score model. Patients were stratified into high-risk (n = 70) and low-risk groups (n = 71) based on the median cutoff values **(** **Figure 6A** **)**. KM curves showed that the odds of developing NPDR were higher in the high-risk group than in the low-risk group, and that the risk of developing NPDR accelerated with a longer history of diabetes **(** **Figure 6B** **)**. The predictive performance of the risk score for NPDR was assessed using a time-dependent ROC curve, with the AUC reaching 0.803 at 10 years, 0.858 at 20 years, and 0.862 at 30 years **(** **Figure 6C** **)**.

### 3.6 Independent prognostic value of eight characteristic metabolites

**Figure 7:**
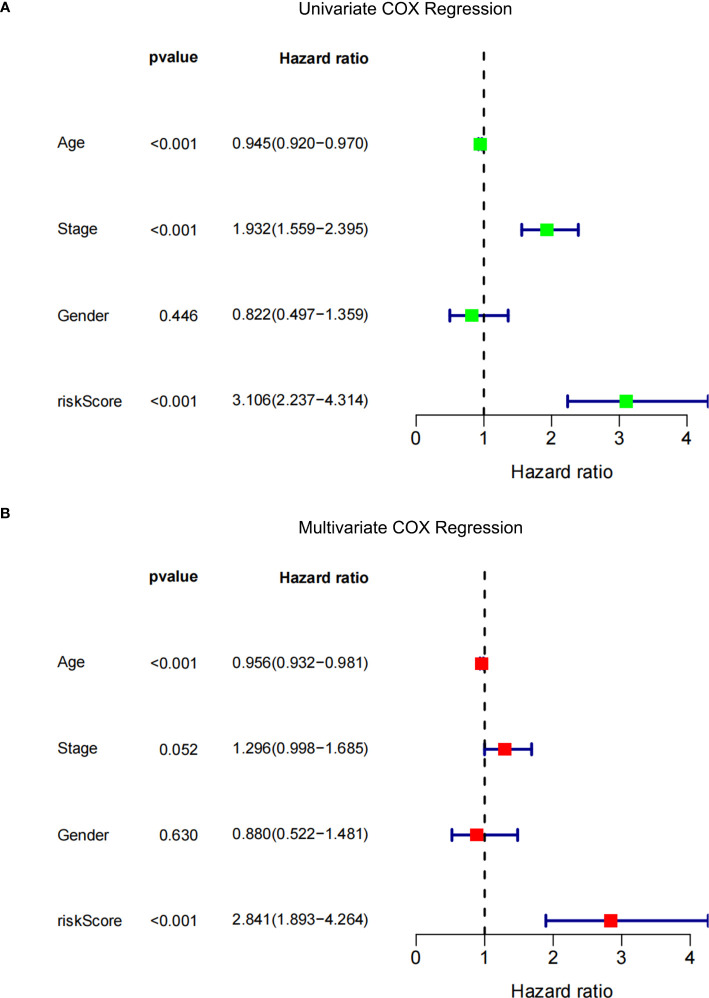
Forest plot. **(A)** Univariate Cox regression analysis of NPDR risk score and clinical characteristics. **(B)** Multivariate Cox regression analysis of NPDR risk scores and clinical characteristics.

Univariate and multivariate Cox regression analyses were performed among the available variables to determine whether the risk score was a predictor of an independent prognosis for NPDR. In the univariate Cox regression analysis, the risk score was significantly associated with NPDR (hazard ratio [HR]=3.106, 95% CI=2.237‒4.314, *P*<0.001) **(** **Figure 7A** **)**. After correcting for other confounders, risk score still proved to be an independent predictor of NPDR in the multivariate Cox regression analysis (HR=2.841, 95% CI=1.893‒4.264, *P*<0.001) **(** **Figure 7B** **)**.

## 4 Discussion

In this study, we developed a risk-score model that can independently predict NPDR using plasma untargeted metabolomics. The risk score was composed of eight metabolites, of which indolelactic acid, thA, 4-dodecylbenzenesulfonic acid, and Arg were protective factors for NPDR, whereas phenylacetylglycine, tiglylglycine, L-aspartic acid, and 3-sulfinato-L-alaninate were risk factors.

DR is divided into two stages: NPDR and PDR. NPDR is divided into three stages according to the severity of the disease (21). The common clinical manifestations of NPDR include microaneurysms, venous beading, and intraretinal microvascular abnormalities. As the disease progresses, NPDR can develop into PDR (22). An untargeted metabolic study in China found that pyruvate, aspartate, glycerol, and cholesterol were differentially expressed in the plasma of PDR and NPDR patients (23). Another untargeted metabolomics analysis found that fumaric acid, uridine, acetic acid, and cytidine can differentiate between NPDR and PDR (24). In addition, a US study found that arginine- and citrulline-related metabolic pathways were abnormal in DR and that fatty acid metabolism is altered in patients with PDR compared with those with NPDR (25).The metabolites screened in this study differed significantly from those reported in other studies, which may be related to the following reasons. First, different experimental and control groups were used. In the current study, NPDR was the experimental group, and DM (without DR) was the control group. Most other studies used NPDR as the control group and PDR as the experimental group. Second, the samples were different. In this study, plasma samples were chosen. Third, different analytical methods were used. The present study used untargeted metabolomics analysis, while some other studies used targeted metabolomics analysis.

Indolelactic acid is a tryptophan metabolite found in human plasma, plasma, and urine. Tryptophan is metabolized by two major pathways in humans, either through kynurenine or *via* a series of indoles, and some of its metabolites are known to be biologically active (26). Indolelactic acid is also a microbial metabolite; urinary indole-3-lactate is produced by clostridium sporogenes (27). Tetracosahexaenoic acid, also known as C24:6N-3 or C24:6omega-3, belongs to the class of organic compounds known as very long-chain fatty acids. 4-dodecylbenzenesulfonic acid is a major component of laundry detergent. Arginine belongs to the class of organic compounds known as l-alpha-amino acids. These are alpha amino acids which have the L-configuration of the alpha-carbon atom. L-Arginine hydrochloride is a drug. Coronary artery endothelial cells from spontaneously diabetic rats were found to have impaired arginine metabolism, which may be associated with diabetic cardiovascular pathology (28). Above all, indolelactic acid, thA and Arg but not 4-dodecylbenzenesulfonic acid,were considered as protective factors for NPDR. A Chinese plasma metabolomics study reported that aspartate was a risk factor for PDR (29). Similarly, we found that aspartate was a risk factor for NPDR, suggesting aspartate plays an essential role in the early and progressive stages of DR. Phenylacetylglycine and tiglylglycine are two kinds of different acyl glycines. Acyl glycines are normally minor metabolites of fatty acids. However, the excretion of certain acyl glycines is increased in several inborn errors of metabolism (30, 31). In certain cases the measurement of these metabolites in body fluids can be used to diagnose disorders associated with mitochondrial fatty acid beta-oxidation. 3-sulfinato-L-alaninate is the organosulfinic acid arising from oxidation of the sulfhydryl group of L-cysteine. It has a role as a metabotropic glutamate receptor agonist, a human metabolite, an Escherichia coli metabolite and a mouse metabolite. It is an organosulfinic acid and a S-substituted L-cysteine. Hence, phenylacetylglycine, tiglylglycine, L-aspartic acid, and 3-sulfinato-L-alaninate were availably identified as risk factors for NPDR.

These metabolites may be affected by certain drugs. Drugs that affect the metabolism of L-aspartic acid may include those that inhibit or induce L-asparagine synthetase or L-asparaginase. For example, methotrexate can inhibit L-asparagine synthetase and reduce the synthesis of L-asparagine from L-aspartic acid2 (26, 27). Tiglylglycine is a metabolite of isoleucine and valine. Tiglylglycine can be elevated in urine of patients with beta-ketothiolase deficiency or with disorders of propionate metabolism (28). Drugs that affect the metabolism of tiglylglycine may include those that interfere with the enzymes involved in the catabolism of isoleucine and valine, such as beta-ketothiolase, propionyl-CoA carboxylase, and methylmalonyl-CoA mutase (29). Indolelactic acid is a metabolite of tryptophan. Drugs that affect the metabolism of indolelactic acid may include those that interfere with the enzymes involved in the biosynthesis and degradation of tryptophan and its metabolites, such as tryptophan hydroxylase, indoleamine 2,3-dioxygenase, kynurenine aminotransferase, and kynureninase. Drugs that induce or inhibit these enzymes may alter the level of indolelactic acid in the body. For example, fluoxetine can inhibit tryptophan hydroxylase and reduce the synthesis of serotonin from tryptophan. On the other hand, interferon-gamma can induce indoleamine 2,3-dioxygenase and increase the catabolism of tryptophan to kynurenine and its derivatives (30, 31). Threonic acid is a sugar acid derived from threose. Threonic acid can also be produced from the metabolism of ascorbic acid (vitamin C) by L-threonate 3-dehydrogenase2. Threonic acid can be further metabolized to glyceraldehyde 3-phosphate and acetyl-CoA by threonate 4-dehydrogenase and threonate aldolase. Drugs that affect the metabolism of threonic acid may include those that interfere with the enzymes involved in the biosynthesis and degradation of ascorbic acid and threonic acid, such as L-threonate 3-dehydrogenase, threonate 4-dehydrogenase, and threonate aldolase. Drugs that alter the level of ascorbic acid or threonic acid may also affect the level of other metabolites in the same pathway. For example, acetaminophen can deplete ascorbic acid and increase the oxidative stress in the body (29, 32–34). Drugs that affect the metabolism of L-arginine may include those that interfere with the enzymes involved in the biosynthesis and degradation of L-arginine and its metabolites, such as arginase, nitric oxide synthase, arginine decarboxylase, argininosuccinate synthase, and argininosuccinate lyase2. Drugs that alter the level of L-arginine or nitric oxide may also affect the level of other metabolites in the same pathway. For example, sildenafil can enhance the effect of nitric oxide by inhibiting its breakdown by phosphodiesterase type 5. On the other hand, methotrexate can inhibit argininosuccinate synthase and reduce the synthesis of L-arginine from L-citrulline (35).

Our study has a few limitations. First, we have not yet validated these plasma metabolites diagnostic efficacy in a cohort. Second, this study included only the yellow race, and the results should be validated in more races.

In conclusion, we identified previously unreported metabolic alterations in patients with NPDR based on untargeted metabolome analysis of venous plasma. We constructed a risk score model for NPDR from seven effective plasma metabolites, including indolelactic acid, thA, Arg, Phenylacetylglycine, Tiglylglycine, L-Aspartic acid, and 3-Sulfinato-L-alaninate.

## Data availability statement

The data supporting the findings of this study are available from the corresponding author ZL upon reasonable request.

## Ethics statement

The studies involving human participants were reviewed and approved by Shenzhen people’s hospital. The patients/participants provided their written informed consent to participate in this study.

## Author contributions

XW, GY, TM and PZ contributed to the acquisition, analysis, and interpretation of data. SY, HS and JD drafted the manuscript or revised it critically for important intellectual content. JL, LK, and ZL analyzed the data and revised the article critically for important intellectual content. SY and XW contributed to the conception and study design. All authors approved the version to be published.
